# N95 masks increase brain blood velocity and parasympathetic outflow, yet worsen orthostatic symptoms in a healthy cohort

**DOI:** 10.14814/phy2.70402

**Published:** 2025-06-18

**Authors:** Tania J. Pereira, Heather Edgell

**Affiliations:** ^1^ School of Kinesiology and Health Sciences York University Toronto Ontario Canada; ^2^ Muscle Health Research Centre York University Toronto Ontario Canada

**Keywords:** brain blood flow, mask wearing, orthostatic intolerance, symptoms, tilt table testing

## Abstract

Mask wearing became commonplace in everyday life during the COVID‐19 pandemic and masks are frequently used in certain professions. Masks can increase end‐tidal CO_2_ and the cerebrovasculature is known to vasodilate in response to hypercapnia. Orthostatic intolerance (OI) is the inability to tolerate postural transitions due to the displacement of blood volume away from the cerebral circulation, and we hypothesized that wearing a mask would improve OI symptoms by increasing brain blood flow. Young, healthy participants (*n* = 27) completed 10 min of 70° head‐up tilt while wearing/not wearing an N95 mask (randomized), while hemodynamics and blood velocity within the middle cerebral artery (MCAv) were measured. Systolic, diastolic, and mean blood pressure and mean and diastolic MCAv decreased during tilt in both conditions (all *p* < 0.05; Table 1). Systolic MCA_V_ was elevated while wearing a mask (*p* < 0.05). Some OI symptoms were exacerbated during the mask trial (all *p* < 0.05) including the overall OI score (*p* = 0.002; Table 2). There appears to be a disconnect between the physiological response to mask wearing and OI symptoms, potentially indicating that there is a psychological component to mask wearing. While we have provided evidence that masking increases brain blood flow, the psychological effects may outweigh potential benefits to the cerebral circulation.

## 1 INTRODUCTION

Due to the COVID‐19 pandemic, mask wearing became integrated into the daily lives of individuals across a variety of professions, specifically healthcare. Researchers began to investigate the physiological demand of prolonged mask wearing. Several studies have found that wearing an N95 or surgical mask increases end‐tidal carbon dioxide (ETCO_2_) in healthy adults (Acuti Martellucci et al., 2022; Brooks et al., 2023; Karsli et al., 2023; Özdemir et al., 2020; Shechtman et al., 2022). Considering that the cerebrovasculature is highly responsive to arterial CO_2_ and that increases in arterial CO_2_ would lead to vasodilation (i.e., an increase in brain blood flow) (Battisti‐Charbonney et al., 2011; Hoiland et al., 2019; Meng & Gelb, 2015; Ogoh et al., 2008), this may suggest that mask wearing could in fact be protective of brain blood flow and potentially improve orthostatic intolerance (OI) or the inability to maintain an upright position or tolerate postural transitions. It is important to consider potential interventions to improve OI as it can lead to dramatic reductions in the ability to work and quality of life. For example, Winker et al. (2003) found that OI was present in roughly a quarter of men in a subset of individuals in military service. Furthermore, in a large survey‐based study in postural orthostatic tachycardia syndrome (POTS), only 48% of over 5000 patients reported employment and over 70% of patients reported lost income due to symptoms (Bourne et al., 2021).

During standing, brain blood flow can be significantly diminished due to a large displacement of blood volume to the lower body, which can result in symptoms of cerebral hypoperfusion (i.e., lightheadedness, fainting, etc.). In occupations requiring prolonged standing periods, the experience of OI symptoms may be detrimental. Thus, mask wearing during orthostatic challenge may be beneficial to reduce the experience of symptoms of OI by increasing brain blood flow. N95s are recommended or required for some professions (e.g., painting, healthcare)—further, during the COVID‐19 pandemic, a much greater number of lay persons were wearing masks for safety. Interestingly, numerous researchers have demonstrated that wearing N95s increases end‐tidal CO_2_ (Acuti Martellucci et al., 2022; Brooks et al., 2023; Karsli et al., 2023; Özdemir et al., 2020; Shechtman et al., 2022), which could influence cerebrovascular and autonomic function and potentially improve orthostatic intolerance (OI). OI is an important factor to consider for all since it could lead to greater occupational health and safety risks. The current study aims to investigate the effect of wearing an N95 mask on cerebral blood velocity at rest and during upright tilt compared to a non‐masked control trial. We hypothesized that wearing the N95 mask would increase end‐tidal CO_2_ and therefore improve brain blood flow indices and symptoms of OI.

## 2 METHODS

The Office of Research Ethics (ORE) at York University approved the ethics application for this study in accordance with the Helsinki declaration of 1975, as revised in 2008. Written informed consent was provided by all participants prior to participating in the study protocol. A convenience sample of 27 healthy individuals aged 18–30 years was recruited to participate from the York University population via word of mouth and poster recruitment (*n* = 10 male, *n* = 17 female). Individuals were excluded for a current diagnosis or previous history of any cardiovascular, cerebrovascular, or autonomic chronic disease, condition, or illness that may influence their physiological responses to tilt. All participants refrained from caffeine, alcohol, strenuous exercise, and fatty foods for at least 12 hours prior to testing.

### 2.1 Experimental protocol

In a randomized order, participants completed two trials of a 70° head‐up tilt test, including 5 min of supine rest and 10 min of tilt in an open‐label design. Participants completed the protocol while either wearing (i.e., masked condition) or not wearing (i.e., control condition) an N95 mask, with a 30‐minute break between trials. Neither participants nor experimenters were blinded to the order of trials. All testing was conducted at York University.

### 2.2 Cardiovascular & cerebrovascular measures

ECG and beat‐to‐beat finger photoplethysmography (BMEye Nexfin, Amsterdam, NL) were used to continuously monitor heart rate (HR) and mean arterial pressure (MAP), respectively. The time and frequency domains of heart rate variability (HRV) were calculated using 5 min of continuous ECG at rest and during the last 5 min of tilt, using the HRV module in the LabChart Pro software (Version 8.1.9, ADinstruments). Time domain variables include the standard deviation of the R–R interval (SDRR), the root mean square of successive differences (RMSSSD), and the percentage of successive differences between RR intervals that are greater than or equal to 50 ms (pRR50). A Hanning window of length 1024 and 50% overlap was used for the frequency domain analysis. Absolute and normalized spectral power in the low frequency (LF) (0.04–0.15 Hz) and high frequency (HF) (0.15–0.45 Hz) were calculated. The hand from which we determined MAP was kept at heart level throughout both trials. Automated pulse contour analysis (i.e., Modelflow algorithm) was used to estimate stroke volume, which was normalized to body surface area (i.e., stroke volume index; SVi). Modelflow has been shown to be accurate with showing changes during orthostatic stress (Lucci et al., 2023). Cardiac output (Qi) and total peripheral index (TPRi) were calculated with HR, MAP, and SVi (i.e., Qi = SVi × HR and TPRi = MAP/Qi). A 2‐MHz Transcranial Doppler (TCD) probe (Multigon Industries Inc.) measured right middle cerebral artery blood velocity (MCA_V_) where the TCD probe was positioned on the right temporal window and fixed by an adjustable headband.

### 2.3 Orthostatic symptom scores

The Vanderbilt Orthostatic Symptom Score was used to quantify various OI symptoms on a scale of 0–10, with 10 indicating higher severity (Raj et al., 2005), at the end of the 10 minutes of tilt while still upright.

### 2.4 Data and statistical analysis

Participant characteristics are described as sample size when categorized into demographical groups or as mean ± standard deviation where numerical responses were provided. Data were acquired at a rate of 1000 Hz through a PowerLab (16/35, ADInstruments) and recorded using LabChart Pro (Version 8.1.9, ADinstruments). Statistical analyses were performed on Systat SigmaPlot software (Version 15.0, Inpixon), where significance was set to *p* < 0.05 a priori. Two‐way repeated measure ANOVAs were used to compare cardio‐ and cerebrovascular responses to tilt while wearing or not wearing an N95 mask with a post hoc Bonferroni correction for multiple comparisons. All data sets were complete. Total and OI symptom scores were compared with one‐way repeated measure ANOVAs between the masked and control conditions. Sex differences were explored in the changes of the cardiovascular, cerebrovascular, and OI symptom scores in response to tilt. No effects of sex were present for almost all variables (*p* > 0.05); thus, males and females were combined in the primary analysis. Effect sizes for two‐way ANOVAs were estimated using partial *η* ^2^ ($\eta_{p}^{2}$), and effect sizes for one‐way ANOVAs were estimated using Cohen's *d*.

## 3 RESULTS

### 3.1 Participant characteristics

Twenty‐seven healthy individuals (males, *n* = 10; females, *n* = 17) participated in the current study. Most participants self‐identified as Asian descent (*n* = 13), followed by Middle Eastern (*n* = 6), Caucasian (*n* = 6), African (*n* = 1) and undisclosed (*n* = 1). Participants were 20 ± 3 years of age, had a body mass of 71 ± 16 kg, and were 166 ± 11 cm in height. Participants engaged in moderate to strenuous exercise 3 ± 1 days per week.

### 3.2 Cardiovascular & cerebrovascular measures

**FIGURE 1:**
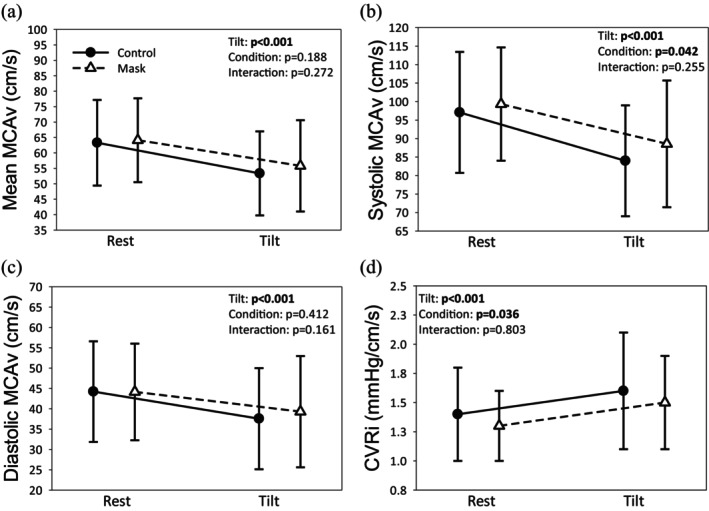
The mean (a), systolic (b), diastolic (c) middle cerebral artery blood velocity (MCAv), and cerebrovascular resistance index (d) response to head‐up tilt while wearing (white triangles) or not wearing (black circles) an N95 mask.

In response to tilt, HR increased while blood pressure (systolic, diastolic, and MAP), SVi, and TPRi decreased in both trials (all *p* < 0.05; Table 1; all large effects $\eta_{p}^{2}$ > 0.28). SDRR, RMSSD, and pRR50 decreased during tilt in both trials (all *p* < 0.001; Table 1; all large effects, $\eta_{p}^{2}$ > 0.89). LF power increased during tilt in both trials (*p* < 0.001; large effect $\eta_{p}^{2}$ = 0.81) and was lower throughout the masked trial compared to the control trial (*p* = 0.047; Table 1; large effect, $\eta_{p}^{2}$ = 0.22). Comparatively, HF power decreased during tilt in both trials (*p* < 0.001; large effect, $\eta_{p}^{2}$ = 0.79) and was higher throughout the masked trial compared to the control trial (*p* = 0.044; Table 1; large effect, $\eta_{p}^{2}$ = 0.22). Qi and LF/HF ratio were unaffected by mask‐wearing or tilt (both *p* > 0.05; Table 1). During tilt, mean (Figure 1a), systolic (Figure 1b), and diastolic MCA_V_ (Figure 1c) decreased, while CVRi (Figure 1d) increased in both trials (all *p* < 0.001; all large effects, $\eta_{p}^{2}$ > 0.58). Regardless of time, systolic MCA_V_ was higher, and CVRi was lower throughout the masked trial compared to control (both *p* < 0.05; both large effects, $\eta_{p}^{2}$ > 0.27).

**TABLE 1:** The cardiovascular and cerebrovascular responses to head‐up tilt while wearing or not wearing an N95 mask.

	Mask		Control		*p*‐Value		
	Rest	Tilt	Rest	Tilt	Mask	Tilt	Mask × tilt
HR (bpm)	69 ± 11	88 ± 10^a^	67 ± 8	88 ± 11^a^	0.336	**<0.001**	0.405
MAP (mm Hg)	84 ± 11	80 ± 12^a^	86 ± 11	81 ± 12^a^	0.244	**0.001**	0.185
SBP (mm Hg)	114 ± 16	105 ± 18^a^	112 ± 21	102 ± 18^a^	0.425	**<0.001**	0.532
DBP (mm Hg)	71 ± 10	69 ± 11^a^	79 ± 19	74 ± 19^a^	0.056	**0.011**	0.081
Qi (L/min)	6.6 ± 1.4	6.5 ± 1.6	6.7 ± 1.4	6.8 ± 1.5	0.341	0.923	0.259
SVi (mL/m^2^)	97 ± 21	75 ± 18	99 ± 19	77 ± 16	0.092	**<0.001**	0.847
TPRi (mm Hg/L/min/m^2^)	13.3 ± 4.1	12.9 ± 3.4	13.6 ± 3.5	12.5 ± 3.2	0.858	**0.026**	0.134
SDRR (ms)	81 ± 22	52 ± 18^a^	75 ± 29	51 ± 20^a^	0.249	**<0.001**	0.122
RMSSD (ms)	78 ± 33	32 ± 14^a^	71 ± 40	31 ± 13^a^	0.197	**<0.001**	0.253
pRR50 (%)	45 ± 20	11 ± 11^a^	38 ± 22	9 ± 9^a^	0.056	**<0.001**	0.063
LF (nu)	39 ± 24^b^	60 ± 21^a,b^	43 ± 19	69 ± 19^a^	**0.047**	**<0.001**	0.222
HF (nu)	60 ± 23^b^	40 ± 21^a,b^	56 ± 19	32 ± 18^a^	**0.044**	**<0.001**	0.225
LF/HF ratio	2.71 ± 7.55	3.54 ± 5.68	1.15 ± 1.39	4.19 ± 4.84	0.600	0.080	0.246

*Note*: All values are mean ± SD. Significance is represented by bold text (*p* < 0.05). a represents significantly different than rest (*p* < 0.05). b represents significantly different than control (*p* < 0.05).

Abbreviations: DBP, diastolic blood pressure; HF, high frequency; HR, heart rate; LF, low frequency; MAP, mean arterial pressure; pRR50, proportion of RR interval differences greater than 50 ms; Qi, cardiac output index; RMSSD, root mean square standard deviation; SBP, systolic blood pressure; SDRR, standard deviation of RR intervals; SVi, stroke volume index; TPRi, total peripheral resistance index.

Males had a greater drop in diastolic MCA_V_ due to tilt during both trials (male, control −9 ± 6 cm/s, masked −7 ± 4 cm/s vs. female, control −5 ± 6 cm/s, masked −3 ± 4 cm/s; *p* < 0.05), but there was no effect of wearing a mask, and no other variables were different between the sexes (all *p* > 0.05).

### 3.3 Vanderbilt orthostatic intolerance scores

Symptoms of nausea (*p* = 0.034, *d* = 0.55), rapid heartbeat (*p* = 0.011, *d* = 0.43), and shortness of breath (*p* = 0.001, *d* = 0.90) were scored higher during the masked trial than the control trial (Table 2). Additionally, the Total Vanderbilt Orthostatic Symptom Score was higher in the masked trial compared to the control trial (*p* = 0.002, *d* = 0.55; Table 2). Blurred vision, chest discomfort, headache, lightheadedness, mental cloudiness, and tremulousness were not different between the trials (all *p* > 0.05; Table 2).

**TABLE 2:** Orthostatic intolerance symptom scores during mask and control trials.

	Mask	Control	*p*‐Value
Blurred vision	1 ± 2	1 ± 1	0.285
Chest discomfort	1 ± 2	1 ± 2	0.414
Headache	2 ± 3	1 ± 2	0.058
Lightheadedness	3 ± 3	2 ± 2	0.090
Mental cloudiness	3 ± 2	2 ± 2	0.099
Nausea	1 ± 2*	0 ± 1	**0.034**
Rapid heartbeat	3 ± 3*	2 ± 2	**0.011**
Shortness of breath	4 ± 3*	1 ± 2	**0.001**
Tremulousness	2 ± 2	1 ± 2	0.052
Total Score	20 ± 17*	12 ± 12	**0.002**

*Note*: All values are mean ± SD. Significance is represented by bold text and * (*p* < 0. 05).

## 4 DISCUSSION

The major findings of the current study are that mask wearing increased systolic MCA_V_ and decreased CVRi at rest and during tilt while changing the autonomic control of heart rate; however, cerebrovascular and hemodynamic responses were not different during tilt between the masked and control conditions. Unexpectedly, wearing a mask exacerbated some OI symptoms and increased the total OI symptom score in response to tilt compared to the control condition. There seems to be a disconnect between the physiological responses to tilt and the symptom experience, considering that masking increased systolic cerebral blood flow yet also induced greater OI symptom severity— which may indicate that these symptoms were psychologically induced.

As expected, blood pressure, brain blood flow indices, and parasympathetic control of HR (frequency domain analysis) decreased during upright posture regardless of mask wearing (Kim et al., 2011; Robertson et al., 2020; van Campen et al., 2018). Wearing the mask (regardless of posture) increased parasympathetic activity as evidenced by the increased HF power of heart rate variability. Simultaneously, LF control of HR was reduced while wearing the mask, yet there was no effect on the LF/HF ratio, a marker of sympathetic activity. Therefore, since LF is a combination of sympathetic and parasympathetic activity and potentially indicates baroreflex sensitivity (Goldstein et al., 2011; Rahman et al., 2011), mask wearing could be influencing blood pressure control via the parasympathetic control of HR. Taken together, our observations suggest a shift of cardiac autonomic control towards parasympathetic dominance during the masked trial. Future studies should consider blood pressure variability analysis and detrended fluctuation analyses due to the non‐stationarity of the data during orthostatic stress.

There is growing evidence of a parasympathetic influence on brain blood flow, such that vagal nerve stimulation improves brain blood flow (Conway et al., 2006). Furthermore, while we did not directly measure ETCO_2_, masking has been previously shown to increase ETCO_2_ (Acuti Martellucci et al., 2022; Brooks et al., 2023; Karsli et al., 2023; Özdemir et al., 2020; Shechtman et al., 2022), and hypercapnia is a known potent cerebrovascular dilator (Coverdale et al., 2015; Hoiland et al., 2019; Verbree et al., 2014). Therefore, the observed increase in systolic MCAv while masked could potentially be due to either hypercapnia or greater parasympathetic activation. Burma and colleagues (Burma et al., 2024) observed that the largest hypercapnic vasodilatory response occurred during systole in the middle cerebral arteries, which may be partly due to the increasing acidic microenvironment of the cerebrovasculature (Liu et al., 2019) and increased shear stress that could contribute to increased vasodilation during systole (Balligand et al., 2009). Furthermore, mild hypercapnia has been shown to enhance the increase in cerebral blood flow caused by parasympathetic stimulation in rodent models (Morita et al., 1994). In the current study, mask wearing more effectively maintained blood flow during tilt as systolic MCA_V_ was higher and CVRi was lower through the whole trial; however, these differences were small. It is unlikely that these small differences would be clinically relevant as cerebral blood velocity falls by roughly 25%–50% during tilt in individuals with OI (Harms et al., 2000; Jacob et al., 1999; Novak, 2016). It is important to note that these small changes in brain blood flow indices may have been insufficient to improve OI symptoms. Moreover, these participants were healthy with extremely few symptoms at baseline. Therefore, future studies should investigate participants that already have OI to determine a potential clinical benefit.

Indeed, the most surprising finding was that individuals scored OI symptoms more severely during the masked condition than during the control trial despite the aforementioned increased systolic MCAv and parasympathetic control of HR with lower CVRi. Symptomology may have therefore been influenced by the fact that individuals may find mask wearing psychologically uncomfortable, restrictive, or warm leading to more breathing difficulties (Martin et al., 2020). Furthermore, pain/discomfort has been shown to increase orthostatic symptoms if not overt syncope (Adamec et al., 2013; Novak & Novak, 2025) in some individuals. It is interesting to note that the increase of symptoms noted with masking was more respiratory in origin rather than what might be expected due to changes in brain blood flow. If participants experienced greater anxiety during the masked trial, it is possible that participants may have been hyperventilating (Suess et al., 1980), which could be interpreted as increased dizziness or lightheadedness (Tavel, 2021). Most physiological parameters were similar during both the masked and control conditions; thus, it is unlikely that participants were hyperventilating, or perhaps, it was too subtle for observable physiological differences.

### 4.1 Limitations

Importantly, respiratory rate, tidal volume, ventilation, and ETCO_2_ were not measured due to technical limitations and the necessity of maintaining a proper face seal on the N95 mask. However, numerous other studies have demonstrated that mask wearing increases ETCO_2_ by roughly 0.5–6 mm Hg (Acuti Martellucci et al., 2022; Brooks et al., 2023; Karsli et al., 2023; Özdemir et al., 2020; Shechtman et al., 2022); therefore, we surmise that similar changes in CO_2_ occurred during the current study. It has been previously observed that a roughly 3% decrease in MCA_V_ is associated with a 1 mm Hg drop in ETCO_2_ (Cigada et al., 2000; Ide et al., 2003; Kastrup et al., 1997). In our trials, there was a drop of approximately 13% MCAv due to tilt in the control trial and a drop of approximately 10% MCAv due to tilt in the masked trial; therefore, a slight increase of ETCO_2_ may have occurred in the masked trial, which could have contributed to sparing MCAv during upright tilt. Future studies should consider higher sample sizes using transcutaneous CO_2_ measurements to be able to accurately reflect the relationship between changes in circulating CO_2_ and brain blood flow indices. It is important to note that the findings in the current study may not be generalized to all mask types, and future studies should also include surgical masks. The absence of data on respiratory rate is an important limitation to note since respiratory rate is known to influence the HF aspect of HRV analysis. However, a recent study has found that wearing an N95 mask does not influence respiratory rate, but it increases ventilation and the rating of perceived exertion (Rothstein et al., 2025). Therefore, we suggest that our masked participants may have experienced an unmeasured increase of tidal volume which could be increasing their HRV. As we aimed to maintain the seal of the N95 mask, we were unable to adequately measure tidal volume using a pneumotach. We suggest that future studies consider standing tests during whole body plethysmography.

Limitations exist with measuring cerebral blood flow using transcranial Doppler, as this methodology assumes that the arterial diameter does not change while measuring velocity, and thus, changes in velocity are interpreted as differences in cerebral blood flow. Previous literature has observed that cerebrovascular diameter is relatively stable during changes in ETCO_2_ of less than 7.5 mm Hg (Verbree et al., 2014); thus, it is unlikely that the assumed drop in ETCO_2_ due to upright tilt (~3–4 mm Hg) influenced vessel diameter in the current study. Given that both masking and tilt only cause small changes in ETCO_2_, then it is likely that cerebrovascular diameter was maintained. Future research should more accurately measure changes in brain blood flow using functional MRI or internal carotid blood flow using duplex ultrasound to more accurately determine the effect of mask wearing on cerebral blood flow.

An important consideration is that we did not control for, or quantify, how comfortable participants were while wearing masks. Claustrophobia is a significant predictor of mask avoidance and negative attitudes about mask wearing, which are both associated with low adherence to masking mandates (Kılıç et al., 2022). If participants had negative views of mask wearing or felt claustrophobic during the masked condition, this may have added additional psychological stress that influenced their perception of the masked condition. Future research should quantify individual perceptions of masking prior to initiating a masked protocol. Importantly, the results of this study are also limited to healthy populations and should be further explored for potential benefits in clinical populations that experience greater cerebral hypoperfusion during postural changes (Coelho et al., 2024; Del Pozzi et al., 2014; Fraser et al., 2015; Mankovsky et al., 2003). Furthermore, the current study is not sufficiently powered to investigate each ethnic group separately in order to draw conclusions on the influence of race on cerebral perfusion and the potential benefits of mask wearing. This is of note considering that certain ethnic groups may be less tolerant of orthostatic challenges (Hinds & Stachenfeld, 2010). Interestingly, previous work done in the Edgell lab has shown that 3–5 min of tilt table testing to 70° has similarly reduced MAP in young healthy participants (Hazlett & Edgell, 2018; Robertson et al., 2020). Our participant population at York University in Toronto, ON, Canada, is a very ethnically diverse group, and thus, ethnicity could indeed be playing a role in our results and should be recorded in future studies.

Lastly, we could not blind the participants to the order of trials (due to the nature of wearing a mask or not), and researchers were similarly not blinded. Furthermore, the informed consent document did inform participants of the study hypothesis. While we randomized the trials to hopefully minimize an order effect, we cannot state with certainty that an absence of blinding did not bias our results.

## 5 CONCLUSION

While mask wearing marginally increased systolic cerebral blood velocity and parasympathetic outflow in healthy, young adults, with a reduction of cerebrovascular resistance, this improvement did not translate to benefits in OI symptoms. Participants scored their experience of OI symptoms more severely and had a higher overall total OI symptom severity score in response to tilt while masked, compared to non‐masked. These symptoms may be psychologically induced given that some individuals may find mask wearing uncomfortable due to feelings of restricted breathing or trapped heat. These perceptions may outweigh the potential physiological benefits of mask wearing on brain blood flow; however, these conclusions cannot be drawn in clinical populations nor in individuals who are more comfortable wearing masks (i.e. healthcare professionals, etc.). Future research should consider the psychological influence of mask wearing and how this perception may impede physiological benefits in healthy and clinical populations.

## AUTHOR CONTRIBUTIONS

Conceptualization—TJP and HE; data curation—TJP and HE; formal analysis—TJP and HE; funding acquisition—HE; investigation—TJP; methodology—TJP and HE; project administration—TJP and HE; resources—HE; software—TJP and HE; supervision—HE; validation—TJP and HE; visualization—TJP; writing—original draft—TJP; writing—review and editing—TJP and HE.

## FUNDING INFORMATION

NSERC awarded to HE, OGS and Susan Mann Dissertation Scholarship awarded to TJP.

## CONFLICT OF INTEREST STATEMENT

None declared.

## Data Availability

The data that support the findings of this study are available on request from the corresponding author. The data are not publicly available due to privacy or ethical restrictions.
